# Adenoma and Polyp Detection Rates in Colonoscopy according to Indication

**DOI:** 10.1155/2017/7207595

**Published:** 2017-12-27

**Authors:** Erika S. Boroff, Molly Disbrow, Michael D. Crowell, Francisco C. Ramirez

**Affiliations:** ^1^Division of Gastroenterology, Mayo Clinic, Scottsdale, AZ, USA; ^2^Department of Medicine, Mayo Clinic, Scottsdale, AZ, USA

## Abstract

**Background:**

Adenoma detection rate (ADR) is a validated quality measure for screening colonoscopy, but there are little data for other indications. The distribution of adenomas is not well described for these indications.

**Aim:**

To describe ADR and the adenoma distribution in the proximal and distal colon based on colonoscopy indication.

**Methods:**

Outpatient colonoscopies are subdivided by indication. PDR and ADR for the entire colon and for proximal and distal colon. Data were compared using generalized estimating equations to adjust for clustering amongst endoscopists while controlling for patient age and gender.

**Results:**

3436 colonoscopies were reviewed (51.2%: men (*n* = 1759)). Indications are screening 49.2%, surveillance 29.3%, change in bowel habit 8.4%, bleeding 5.8%, colitides 3.0%, pain 2.8%, and miscellaneous 1.5%. Overall ADR was 37% proximal ADR 28%, and distal ADR 17%. PDR and ADR were significantly higher in surveillance than in screening (PDR: 69% versus 51%; ADR: 50% versus 33%; *p* = 0.0001). Adenomas were more often detected in the proximal than in the distal colon, for all indications.

**Conclusions:**

Prevalence of polyps and adenomas differs based on colonoscopy indication. Adenoma detection is highest in surveillance and more commonly detected in the proximal colon. For quality assurance, distinct ADR and PDR targets may need to be established for different colonoscopy indications.

## 1. Introduction

Colorectal cancer (CRC) is the third leading cause of cancer-related mortality for men and women in the United States [1]. In 2014, over 136,000 new cases of CRC were expected to be diagnosed, and over 50,000 people would have died from this condition. Screening colonoscopy with polypectomy has been associated with a reduction in the incidence of colorectal cancer [2–4] as well as mortality secondary to CRC [5]. Published guidelines recommend screening colonoscopy for all average-risk, asymptomatic adults [6, 7]. The effectiveness of colonoscopy to reduce CRC risk is dependent on the ability of the endoscopist to detect and remove adenomatous polyps. A growing acknowledgement of variable adenoma detection amongst endoscopists has led to increasing calls for quality metrics in colonoscopy [8, 9]. Both the US Multi-society Task Force on Colorectal Cancer and the American Society of Gastroenterology have published guidelines for quality colonoscopy, which include a regular accounting and reporting of adenoma detection rates for individual endoscopists [10–12]. Whereas ADR targets have been established for screening colonoscopy, no recommendations exist for colonoscopies performed for other indications. It has been observed that adenoma detection is increased in surveillance colonoscopy compared with screening. Little is known, however, about the prevalence of adenomas in patients undergoing colonoscopy for other indications. In addition, the most common location for CRC is the proximal colon [1]. Historically, screening colonoscopy has demonstrated less protection from proximal colon cancers compared with distal lesions [13, 14]. We sought to identify whether adenoma detection and the distribution of adenomas differ based on colonoscopy indication. We inquire if ADR targets for common colonoscopy indications should be developed, given the majority of colonoscopies performed are for indications other than screening.

## 2. Methods

### 2.1. Study Design

We performed a retrospective review of a colonoscopy database maintained by Mayo Clinic in Scottsdale, Arizona. The local institutional review board granted exemption from informed consent as patients were receiving the standard of care without reference to any study, data collection did not influence medical practice, and data was deidentified before analysis.

### 2.2. Patients

Data of patients over 50 years of age, scheduled for a nonurgent, outpatient colonoscopy between October 1, 2010, and August 30, 2012, were included.

### 2.3. Endoscopic Equipment

All examinations were performed using high-definition colonoscopies (Olympus PCF-Q180AL, CFQ180AL; Olympus America, Center Valley, PA).

### 2.4. Data Collection

Colonoscopies were performed at a single academic medical center by 21 experienced, board-certified gastroenterologists over 22 months of data collection. The standard of care of our institution is to recommend a clear liquid diet for patients on the day prior to the colonoscopy and to utilize a split-dose bowel preparation of either polyethylene glycol or sodium phosphate, where half of the preparation was taken on the day of the procedure. Patients who had early appointments are given the option of a single dose preparation given the evening before the colonoscopy.

Patient demographic information, study indication and presence, location, and number of polyps identified per each colonoscopy were recorded. Demographic information was limited to age and gender at the time of colonoscopy.

Study indications were grouped into the following categories: screening, surveillance, gastrointestinal bleeding, change in bowel habit, colitides, pain, or miscellaneous. Screening colonoscopy was limited to those patients without prior colonoscopy or with no prior history of adenomatous polyps identified on previous screening colonoscopy. Surveillance studies were performed for those patients with prior adenomatous colon polyps identified on prior colonoscopy. The indication of gastrointestinal bleeding (GIB) included patients with history of overt bleeding, occult bleeding identified via a guaiac-based fecal occult blood test (gOBT) (Hemoccult II or Hemoccult Sensa; Beckman Coulter Inc., Brea, CA), or anemia.

The indication of “change in bowel habit” included the indications of diarrhea and constipation. Colitides encompassed patients with known inflammatory bowel disease or other colitis defined per the discretion of the referring physician. The indication of pain included patients referred for colonoscopy due to abdominal, pelvic, or rectal pain. Miscellaneous indications included colonoscopy performed for reasons other than the indications listed above.

Polyp location was described as “proximal” or “distal” depending on the polyp's relationship to the splenic flexure.

To obtain information regarding adenomatous polyps, a retrospective view of the electronic medical record for pathology data was performed. All biopsy specimens in our institution are reviewed by pathologists specialized in gastrointestinal pathology.

### 2.5. Measurements

We subdivided the total number of colonoscopies by indication and then calculated the adenoma detection rate (ADR), the polyp detection rate (PDR), the proximal ADR, defined as the prevalence of patients with at least one adenoma detected proximal to the splenic flexure, and the distal ADR, defined as the prevalence of patients with at least one adenoma detected distal to the splenic flexure, for the group of gastroenterologists.

### 2.6. Statistical Analysis

All statistical analyses were completed using SAS (SAS System for Windows, version 9.2; SAS Institute Inc., Cary, NC) or IBM SPSS (SPSS version 22; Chicago, IL). Continuous data were presented as mean ± standard deviation (SD) or [95% confidence intervals (CI)]. Categorical data are summarized as frequencies and percentages. Generalized estimating equations were used to adjust for nonindependence within endoscopist clusters for procedural level data. The differences between continuous variables were assessed using Student *t*-tests. The chi-squared test was used to assess differences in distributions of categorical variables. Results were considered statistically significant for a (2-tailed) *p* value of <0.05.

## 3. Results

A total of 3436 colonoscopies were performed during the study period. The average patient age was 62.5 years (±10.5 years), and 51.2% of the patients were men (*n* = 1759). Patient demographics and colonoscopy indications are described in Table 1. Screening colonoscopies accounted for 49.2% of all studies in the cohort (*n* = 1690), whereas surveillance colonoscopy comprised 29.3% of all studies (*n* = 1008). Other indications comprised 21.5% (*n* = 738) of colonoscopies. A change in bowel habit represented 8.4% (*n* = 287), gastrointestinal bleeding represented 5.8% (*n* = 200), the colitides 3.0% (*n* = 103), pain in 2.8% (*n* = 96), and miscellaneous indications were listed for 1.5% (*n* = 52).

**Figure 1:**
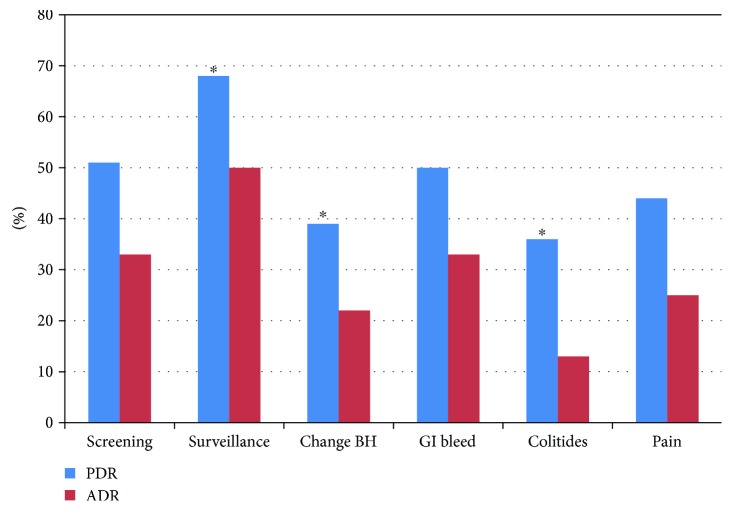
ADR and PDR by indication. A bar graph is depicted comparing the adenoma detection rate (ADR) and polyp detection rate (PDR) for colonoscopies performed for the various indications of screening, surveillance, change in bowel habit, GI bleeding (GIB), colitides, and pain. The legend should state the following: ^∗^significantly differs from screening PDR and ADR. Surveillance ADR and PDR, *p* < 0.001; change in bowel habit ADR and PDR, *p* < 0.001; colitides PDR, *p* = 0.002, and ADR, *p* < 0.001.

The mean PDR for the group was 55%, and the mean ADR was 37% similar to previously published data and in keeping with current quality standards (Table 2). Polyp detection and adenoma detection were significantly higher for surveillance colonoscopy compared with screening colonoscopy, even when controlling for age and patient gender: [OR 2.11 (95%CI 1.79–2.4); *p* < 0.0001] for PDR and [OR 2.02 (95%CI 1.73–2.37); *p* < 0.0001] for ADR (Figure 1). In addition, the mean number of adenomas detected per patient (MAP) was higher in surveillance colonoscopy compared with screening studies (MAP 1.15 versus 0.60; *p* = 0.0001). In comparison with the ADR associated with screening colonoscopy, the ADR was significantly lower when colonoscopy was performed for other indications, such as a change in bowel habit [OR 0.56 (95%CI 0.42–0.76); *p* < 0.001] or for evaluation of a patient with colitis [OR 0.29 (95%CI 0.16–0.52; *p* = 0.0001]. ADR was also lower when the indication for colonoscopy was pain, although this finding did not reach statistical significance. Patients who completed colonoscopy for a change in bowel habit or for pain were more likely to be women (66% and 60% versus 49% in screening; *p* < 0.001 and *p* = 0.001) (Table 3); however, the differences in PDR and ADR persisted when controlling for age, gender, and clustering of data amongst endoscopists. In cases where prior colonoscopy data was available, the average interval between screening colonoscopies was 6 ± 1.4 years. In contrast, surveillance colonoscopy was performed at 4 ± 2 years (Table 4). Colonoscopy ordered for a change in bowel habit or for abdominal pain occurred at 5 ± 2 years, whereas those patients with colitides underwent colonoscopy at shorter intervals on average, 3 ± 1.8 years, and GI bleeding at 4 ± 2.4 years.

**Figure 2:**
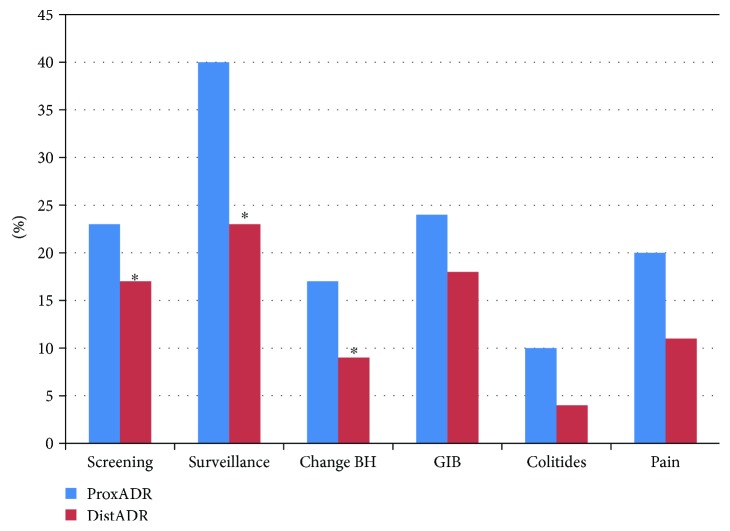
Proximal and distal ADR by colonoscopy indication. A bar graph is depicted comparing the adenoma detection rate from proximal colonic segments (proximal ADR) and distal colonic segments (distal ADR) for the various indications of screening, surveillance, change in bowel habit (BH), GI bleeding (GIB), colitides, and pain. The legend should state the following: ^∗^significantly differs between proximal and distal segments. Screening proximal ADR 20% versus distal ADR 11%, *p* < 0.001; surveillance proximal ADR 40% versus distal ADR 23%, *p* = 0.001; change in bowel habit (BH) proximal ADR 17% versus distal ADR 9%, *p* = 0.005; gastrointestinal bleeding (GIB) proximal ADR 24% versus distal ADR 18%, *p* = 0.154; colitides proximal ADR 10% versus distal ADR 4%, *p* = 0.109; pain proximal ADR 20% versus distal ADR 11%, *p* = 0.144.

Regardless of indication for colonoscopy, adenomas were more often detected in proximal segments compared with distal segments for all indications (Table 2) (Figure 2). For screening colonoscopy, the proximal ADR was 20% and distal ADR was 11% (*p* < 0.001). For surveillance colonoscopy, the proximal ADR was 40% while distal ADR was 23% (*p* = 0.001). For colonoscopy performed for a change in bowel habit, the proximal ADR was 17% compared with 9% for distal ADR (*p* = 0.005). Similarly, the proximal ADR exceeded distal ADR for the indications of bleeding and the colitides, although these values did not reach statistical significance.

## 4. Discussion

We describe the relative frequencies of various indications for nonurgent outpatient colonoscopy performed at single academic medical center. Roughly half of all colonoscopies were completed for screening purposes (49.2%, *n* = 1690), whereas nearly one-third were performed for surveillance (29.3%, *n* = 1008). The remaining 21.5% of colonoscopies (*n* = 738) were completed for indications other than screening or surveillance, the most common being an evaluation of a change in bowel habit, followed by gastrointestinal bleeding and anemia.

The adenoma detection rate for the group was 37%, which exceeds current quality standards. The prevalence of adenomas differed significantly depending on colonoscopy indication. As has been demonstrated in previous studies [15, 16], surveillance colonoscopy was associated with higher rates of adenoma detection when compared with patients who completed colonoscopy for screening purposes (ADR 50% versus 33%; *p* = 0.0001). Conversely, adenoma detection rates for colonoscopy performed for the indications of pain, a change in bowel habit, or evaluation of patients with chronic colitides were significantly lower than that observed for screening studies (ADR 25%, 22%, and 13% for such indications compared with 33% for screening colonoscopy), although these patients underwent colonoscopy at earlier intervals compared with the screening population which may in part explain the decrease in subsequent adenoma detection. Interestingly, adenoma detection rates for outpatient nonurgent colonoscopies performed for bleeding or anemia did not differ from the ADR of screening colonoscopy.

As was demonstrated in our cohort, surveillance colonoscopy has been associated with higher rates of adenoma detection compared with screening colonoscopy [4, 16–18]. Published cohort studies and a meta-analysis have illustrated that patients with three or more adenomas detected with low-grade dysplasia, or one more adenomas with advanced histology detected on index colonoscopy, carry a significant increase in the risk of subsequent adenomas detected on surveillance colonoscopy, as well as the diagnosis of interval colorectal cancer [15, 19, 20]. Based on this information, a shorter surveillance interval has been recommended for those patients in whom greater than 3 adenomas are detected on screening colonoscopy or in whom an adenoma with advanced histology is detected [16, 17].

Furthermore, it has been suggested that separate ADR targets for surveillance and screening colonoscopy be established, as the prevalence of adenomas differs significantly between these populations, although this practice has not been incorporated into CRC screening guidelines [17, 18]. Our data would support such a recommendation.

We observed lower ADRs in colonoscopies performed for nonscreening, nonsurveillance indications.

One explanation for this finding is the fact that colonoscopies performed for nonscreening purposes may be performed in shorter intervals than what is planned for screening colonoscopies. For example, guideline recommendations for inflammatory bowel disease (IBD) management includes annual or biannual colonoscopy to survey for dysplastic lesions in patients who have carried the diagnosis for at least 8–10 years, as well as to assess for mucosal healing [21, 22], which may reduce adenoma detection on subsequent colonoscopies. Also, clinicians may be more inclined to repeat colonoscopy in their IBD patients to evaluate new or persistent symptoms. Another factor may be the degree to which the endoscopist clears the colon of all polyps when the colonoscopy indication is for symptoms such as pain or bleeding, rather than for CRC screening or surveillance. A nonmalignant appearing diminutive polyp may be left in situ if the endoscopist determines the lesion to be noncontributory to the patient's symptoms or signs. Multiple studies have confirmed lower adenoma prevalence in women compared with men [12, 23, 24]. In our cohort, patients undergoing colonoscopy for the indications of a change in bowel habit or for abdominal pain were more commonly women (Table 3). These indications were associated with lower rates of adenoma detection, even when controlling for age and gender, which may represent a higher percentage of patients with functional gastrointestinal disorders compared with the general population or the adenoma surveillance population.

Interestingly, adenomas were more often detected in proximal colonic segments compared with distal segments, regardless of colonoscopy indication, as demonstrated by higher proximal ADRs (ProxADR).

Multiple other studies have demonstrated the same finding [23, 25–27]. As more adenomas are detected in proximal colonic segments, guidelines for quality colonoscopy should continue to advocate for meticulous evaluation of the colonic mucosa with a focus on the proximal colon in particular.

Our study has certain strengths and limitations. The use of a community-based cohort without exclusion of patients based on comorbid illnesses or prior colonoscopy may better reflect the prevalence of adenomas observed in general practice. Pathology reports for all polyps sent for histology were available for review within the same electronic medical record, providing complete pathology data for this cohort of patients. In addition, all pathology specimens were analyzed by pathologists specializing in gastrointestinal illnesses. A limitation of our study includes the collection of data from one institution, which may limit the generalizability of study findings. Data collected did not include information regarding the patients' ethnicity, use of tobacco, body mass index, or family history of adenomas, which may affect the prevalence of colonic neoplasm in the cohort. Further study utilizing data from multiple centers serving patients with diverse backgrounds may be needed to confirm our findings.

We found that adenoma detection rates differ significantly based on colonoscopy indication, with higher prevalence of adenomas in surveillance colonoscopy compared with screening and higher prevalence of adenomas in screening compared with colonoscopy for the colitides, for change in bowel habit or for pain.

We support other authors' recommendations for different ADR targets for screening and surveillance colonoscopy. ADR targets for men and women should also differ, reflecting the difference in the prevalence of adenomas in these populations. Given that adenomas are detected more commonly in proximal colonic segments regardless of colonoscopy indication, guidelines should continue to focus attention on screening and surveillance of the proximal colon, providing feedback to endoscopists regarding their proximal adenoma detection rates.

## Figures and Tables

**Table 1:** Patient demographics and clinical indications for colonoscopy (*n* = 3436).

Characteristic	Number (%)
Mean age, years	62.5 (±10.5)

Sex	
Male	1759 (51.2%)
Female	1677 (48.8%)

Indication	
Screening	1690 (49.2%)
Surveillance	1008 (29.3%)
Change in bowel habit	287 (8.4%)
Gastrointestinal bleeding	200 (5.8%)
Colitides	103 (3.0%)
Pain	96 (2.8%)
Miscellaneous	52 (1.5%)

**Table 2:** Quality indicators by colonoscopy indication.

Indicator	Screening	Surveillance	Change in bowel habits	GIB	Colitides	Pain	Miscellaneous
PDR	51%	68%	39%	50%	36%	44%	62%
ADR	33%	50%	22%	33%	13%	25%	48%
Proximal PDR	31%	49%	22%	31%	17%	25%	48%
Distal PDR	35%	46%	24%	34%	25%	30%	35%
Proximal ADR	23%	40%	17%	24%	10%	20%	42%
Distal ADR	17%	23%	9%	18%	4%	11%	13%
Total number of colonoscopies	1690	1008	287	200	103	96	52

PDR: polyp detection rate; ADR: adenoma detection rate; proximal PDR: detection rate of polyps proximal to the splenic flexure; distal PDR: detection rate of polyps distal to the splenic flexure; proximal ADR: detection rate of adenomas proximal to the splenic flexure; distal ADR: detection rate of adenomas distal to the splenic flexure.

**Table 3:** Percent of colonoscopies performed in women for each colonoscopy indication.

Indication	% of women (*n* women/*n* total)
Screening	49% (825/1690)
Surveillance	42% (422/1008)
Change in bowel habits	66% (189/287)
GI bleeding	52% (103/200)
Colitides	51% (53/103)
Pain	60% (58/96)
Miscellaneous	52% (27/52)

**Table 4:** Colonoscopy intervals by colonoscopy indication.

Indication (% of patients with prior colonoscopy on record)	Interval (years) (+SD) between last and current colonoscopy
Change in bowel habits (28.1%)	5 (2.1)
Colitides (51.5%)	3 (1.8)
GI bleeding (35.7%)	4 (2.4)
Pain (30.5%)	5 (2.2)
Screening (17.9%)	6 (1.4)
Surveillance (67.3%)	4 (2)
Miscellaneous (21.6%)	3 (2.8)
